# Unraveling the Enigma of COVID-19 pneumonia and lung diameters: An HR-CT based study

**DOI:** 10.12669/pjms.42.4.13318

**Published:** 2026-04

**Authors:** Madiha Mushtaq, Ambreen Usmani, Ambreen Surti, Syed Hasan Saeed Kazmi

**Affiliations:** 1 Madiha Mushtaq, MBBS, MPhil Anatomy. Master’s in Public Health, Birmingham City University, Birmingham, UK; 2 Ambreen Usmani, MBBS, MPhil, PGD-E, MCPS-HPE, PhD, FCPS (Anatomy). Principal, Jinnah Sindh Medical University, Karachi, Pakistan; 3 Ambreen Surti, MBBS, MPhil, MHPE, FHEA (UK), Aga Khan University, Karachi, Pakistan; 4 Syed Hasan Saeed Kazmi, 3rd Year MBBS Student, Aga Khan University, Karachi, Pakistan

**Keywords:** COVID-19, Chest computed tomography, Intensive care unit, Non-smokers, Smokers

## Abstract

**Objectives:**

The study aimed to assess the association of COVID-19 pneumonia with lung diameters in cases and controls and evaluate the rate of intensive care unit admission and oxygen requirement in COVID-19 patients.

**Methodology:**

A case-control study was conducted on 84 participants aged 40-60 years (42 COVID-19 survivors and 42 healthy controls) over seven months at a public hospital in Karachi, after ethical approval. Cases were subdivided into smokers and non-smokers. High-resolution CT (HRCT) was used to measure sagittal, coronal, and axial diameters of both lungs. Continuous variables were expressed as mean ± standard deviation, while categorical data were shown as frequencies and percentages. Mann-Whitney U and chi-squared tests were used to compare lung diameters and examine associations with hospital stay, ICU admission, and oxygen use.

**Results:**

COVID-19 survivors showed significantly reduced lung diameters compared with controls (p < 0.001). No significant differences were found between smokers and non-smokers (p = 0.156). Oxygen demand was greater among smokers, but ICU admissions did not differ significantly.

**Conclusion:**

COVID-19 pneumonia resulted in reduction of lung diameters, highlighting HR-CT as a useful tool for follow-up assessment. Smoking did not significantly alter lung diameters and ICU admission but was linked to higher oxygen requirements.

## INTRODUCTION

Corona virus disease of 2019 (COVID-19), a highly contagious disease, became a global pandemic and a public health emergency within three months. In December 2019, cough and dyspnea were reported in 42 patients in Wuhan city, China and patients were declared positive for COVID-19.^1^,^2^ The gold standard method for detection of Corona virus is real-time reverse-transcription-polymerase chain reaction (RT-PCR) assay using nasopharyngeal and oropharyngeal swabs.^3^,^4^ Literature suggests that high resolution computed tomography (CT) scan of chest plays a pivotal part and is a sensitive tool for imaging to establish the diagnosis of COVID-19 pneumonia.^5^

Despite limitations, CT is known to detect infiltration of the lungs in asymptomatic stage of the disease.^1^ A study from University of California radiology department, established the sensitivity of CT scan at 86 % and specificity as 81%.^6^ CT scan showed ground-glass appearance with ill-defined, irregular septal thickening of pleura in these patients.^1^ A prospective study conducted on Iraqi population in 2020, showed approximately 50% of the COVID-19 patients exhibited ground glass opacities (GCO) in lungs on CT scan with significant results.^7^ This appearance is one of the earliest and most frequently appearing sign in COVID-19 patients.

CT scan is known for accurate diagnosis and staging of the disease, as viral infiltration and distribution pattern in the lung lobes is a major finding leading to diagnosis and classification of the disease stage.^1^ Studies have observed bilateral distribution of ground glass opacities with consolidation, thickened septa, crazy paving pattern, air within bronchi, bronchiectasis and bronchial wall and pleural layer thickening and fibrosis.^8^,^9^ Thus, cumulatively establishing the reliability of CT scan in diagnosing COVID-19 pneumonia, especially in the early stages. Due to limited literature on the utility of CT scan to measure lung diameters in COVID-19 patients with pneumonia and its comparison with healthy individuals; this study aimed to assess the association of COVID-19 pneumonia with lung diameters in cases and controls and evaluate the rate of intensive care unit (ICU) admission and oxygen requirement in who are smokers and non-smokers.

## METHODOLOGY

This case-control study was conducted on 84 subjects recruited by non-probability convenience sampling for seven months, September 2021 to March 2022, (from radiology department of public hospital in Karachi. Sample size was calculated using OpenEpi with a confidence interval of 95% and included 42 cases (diagnosed with COVID-19 pneumonia three months before induction), between the ages of 40 - 60 years and were discharged from hospital within six months. This sample size was calculated taking into consideration parameters used by Xiaong et al 2021.^10^ Control group (42 subjects) comprised of healthy individuals who came for high resolution computed tomography scan (HRCT) for anesthetic clearance and were between 40 - 60 years. The exclusion criteria comprised of survivors below the age of 40 or above 60 years. Cases were further categorized into two groups, smokers and non-smokers for lung diameter evaluation, duration of hospital stay and ICU admission.

### Ethical Approval:

It was obtained from Bahria University Karachi Campus (ERC 88/2021; dated December 21, 2021).

Adhering to the guidelines of Helsinki Declaration, a written, informed and understood consent was taken after explaining the aims and methodology. Participation was voluntary, without any incentive and repercussion-free withdrawal from the study. Data was anonymized and coded for confidentiality. Sagittal, coronal and axial diameters were measured for right and left lungs in cases and controls, using the Prime Aquilion-160 slice Toshiba CT scanner by the same trained radiologist to ensure standardization and to reduce inter-observer bias. All measurements were performed by using multiplanar reconstruction in line with standard thoracic CT practice.^11^ Measurements, including axial, coronal and sagittal planes and were standardized using anatomical landmarks like carina, lung width at mid-hilar region and anteroposterior dimensions by measuring the pleural boundaries respectively. All data was noted on a pre-designed questionnaire which included age, gender and comorbidities of the participants.

### Statistical analysis:

It was done by statistical package for social science version 23.0. Continuous variables were expressed as mean ± standard deviation while discreet variables were expressed as frequency and percentage. The Mann-Whitney U test was applied to determine the association between lung diameters of cases and controls and between smokers and non-smokers. Chi-squared test was used to evaluate the association between length of hospital stay and ICU admission of smokers and non-smokers.

## RESULTS

Among 42 cases, 30 (71.42%) were males and 12 (28.57%) were females while the control group included 24 (57.14%) males and 18 (42.85%) females. Cases were further equally divided into current smokers (for past 2 - 15 years) and non-smokers based on smoking history of two packs/day (minimum). CT showed shrunken right and left lungs in cases. The coronal diameters of the right and left lungs showed highly significant results as shown in Table-I and II respectively.

**Table-I:** Comparison of sagittal, coronal and axial diameter of right lung between the cases and control group.

	Group	n	Mean (mm)	p-value
*Sagittal diameter*	Case	42	21.62	0.000
	Control	42	63.38	
*Coronal diameter*	Case	42	21.50	0.000
	Control	42	63.50	
*Axial diameter*	Case	42	21.76	0.000
	Control	42	63.24	

**Table-II:** Comparison of sagittal, coronal and axial diameter of left lung between the cases and control group.

	Group	n	Mean (mm)	p-value
*Sagittal diameter*	Case	42	21.50	0.000
	Control	42	63.50	
*Coronal diameter*	Case	42	22.19	0.000
	Control	42	62.81	
*Axial diameter*	Case	42	21.50	0.000
	Control	42	63.50	

Insignificant results were seen on comparison of sagittal (p=0.614), coronal (p = 0.426) and axial diameters (p = 0.106) of right lungs of smokers and non-smokers by using Man Whitney U test. However, coronal (19.93mm) and axial (18.30mm) diameters were less in smokers compared to nonsmokers, who showed measurements of 22.93mm and 24.41mm respectively on right side. Non-significant results were observed in the lung diameters in sagittal (p = 0.156), coronal (p=0.160) and axial plane (p=0.210) on left side. However, mean measurements were lesser in nonsmokers (18.95mm) as compared to smokers (24.30mm) in sagittal planes. Contrarily axial plane measurements were lesser in smokers (19.03) as compared to nonsmokers (23.75).

Only 35% smokers had ICU admission with no significant difference (p = 0.104) between the two groups. This indicated that smoking had no relation to ICU admission in case of COVID-19 survivors. Seventy percent of smokers required oxygen supplementation to maintain the saturation between SpO2 of 92 -96% with significant results as shown in Table-III.

**Table-III:** Oxygen requirement of smokers and non-smokers.

			Smoking		p-value
			Smoker	Non-Smoker	
Oxygen Requirement	Yes	Count	14	9	0.059
		Percentage	70.0%	40.9%	
	No	Count	6	13	
		Percentage	30.0%	59.1%	

## DISCUSSION

This study exhibits a decrease in sagittal, coronal and axial diameters of both right and left lung in COVID-19 survivors. The diameters show a mean difference of 42 mm from controls. Terms like “long COVID” or “chronic COVID” have been used in literature to describe symptoms of dyspnea, fatigue, chest pain, joint pain etc. in patients after three months of COVID -19 hospitalization.^12^ CT findings like fibrosis and ground glass appearance are long term radiological findings which persist for 3-12 months.^13^ Thus, requiring close monitoring in terms of clinical, laboratory and imaging especially by CT scan as compared to radiographs.^14^ A six months follow-up study conducted by Grag et al, on fifty-six COVID-19 survivors showed septal thickening, GCOs, parenchymal bands and consolidation. Cases showed symptoms of cough, fatigue and myalgia while more than fifty percent exhibited symptoms of dyspnea.^15^

Three-month follow-up CT scan in post COVID-19 patients showed an impairment of pulmonary functions. The current study results measure the diameters of lungs in different planes indicate a shrinkage in size of the lungs thereby impacting the lung functions leading to dyspnea especially in 40 - 60-year-old patients. The outcomes of this study corroborate the evidence in literature which suggests that the presence of opacities and fibrotic lung changes lead to symptoms of dyspnea in survivors.^13^

Moreover, literature implies dyspnea and cough as common symptoms at three-month follow-up of COVID-19 patients. These patients also exhibit lung lesions like opacities and fibrosis on CT chest, leading to a decrease in pulmonary function and saturation.^13^ The current study is the first of its kind to measure the sagittal, coronal and axial lung diameters in COVID-19 survivors, indicating a decrease in lung sizes especially in older age groups. The results of this study are in accordance with a study which followed thirty-nine COVID-19 patients where a decrease in pulmonary function with the presence of opacities in lungs was observed.^16^

Literature identifies lung injury with diffusion capacity impairment and psychological illnesses like depression and anxiety to last from several months to years in survivors.^17^ Recently studies have identified longer duration of hospitalization along with respiratory distress in COVID-19 patients.^18^ Therefore, researchers advise pulmonary evaluation three months post discharge for all critical COVID-19 survivors.^13^ Several factors like age, duration of hospital stay, mechanical ventilation, smoking and comorbidities interplay in determining the influence of disease on patient’s health and pulmonary function. Current research however identifies non-significant results between lung diameters of smokers and non-smokers.

Nicotine suppresses immune response by downregulating interferon-7.^19^ Interferons I and III are reduced while angiotensin converting enzyme receptor expression is increased in smokers with chronic obstructive pulmonary disease (COPD).^19^ Literature stresses worse impact and prognosis along with an increased incidence of COVID-19 in smokers.^20^,^21^ A meta-analysis conducted on 174 studies observed an increase in mortality risk, severity of COVID-19 pneumonia and heightened ICU admissions. Thus, pointing towards adverse outcomes in smokers.^20^ An unusual and novel theory was suggested by Changeux et al, the authors observed competitive inhibition between SARS-CoV-2 and nicotinic acetylcholine receptors (nAChR) found on the epithelial lining of lungs.^21^

These receptors are known in literature to regulate the pro-inflammatory cytokines, thus by competitive inhibition, lowering the chances or severity of COVID-19.^21^ This competitive inhibition and reduction in inflammatory cytokines could be the possible reason that no significant difference in lung diameters was observed between COVID-19 patients who were smokers and non-smokers in the current study. Moreover, a study also stated that smoking induced co-morbidities instead of smoking itself could be the reason for increased severity and mortality in COVID-19 patients.^22^

The current study results show that only seven out of 20 patients with smoking history required ICU admission during their hospital stay. A retrospective study conducted in Cairo, identified age and smoking as a risk factor for ICU admission in COVID-19 patients.^23^ Similar results were identified by Reddy et al. who found that smoking, current or former, impacts the severity of COVID-19 disease and leads to ICU admission.^24^ Unfortunately, the current study failed to categorize the smoking history based on pack/year consumption and this could be the reason for difference in study results.

However, OpenSAFELY, an electronic platform of health records in England exhibited a weak correlation between smoking and disease severity of COVID-19 and ICU admission.^24^ These data support our study results. The reason for this similarity could be the fact that nicotine in cigarettes inhibits the release of inflammatory cytokines.

Current study noted significant results between oxygen use and smoking. Literature suggests a confirmed COVID-19 patient, be it smoker or non-smoker, suffers from hypoxia and a decreased oxygen saturation of <95%.^25^ A cross-sectional study in Nepal, identified low levels of oxygen in smokers. This is due to the binding of released carbon monoxide with hemoglobin thus decreasing oxygen saturation in blood.^26^ A study identified that most of its participants required oxygen through nasal cannulas and face masks while few required mechanical ventilations. Chronic smoking can impact saturation levels in individuals and increase the need for oxygen supplementation.^27^

With the aim to identify and timely manage the respiratory conditions, a clinical and radiological assessment is advised 12 weeks post discharge. Having said this, the current study also demonstrates the presence of relevant lung abnormalities on CT scan, thus highlighting the need for follow-up. This study, being the first of its kind conducted in a developing country, highlights the significance of CT scan for follow-up patients.

### Limitations:

Being time bound, single centered and small sampled, makes the results not generalizable. The authors therefore recommend multicenter studies with larger data sets for robust generalizable results.

## CONCLUSION

This study was able to conclude that COVID-19 can cause lung damage which can persist long after the acute symptoms have weaned off. Hence, CT scan plays a significant role in detecting the changes in lung diameters as well as in parenchyma. Smoking though understood as major player in impacting COVID-19 severity, was found to be non-significantly involved in the whole scenario.

### Authors Contribution:

**MM:** Conceived, designed and did statistical analysis & editing of manuscript, is responsible for integrity of research.

**MM, AU, AS & HK:** Did data collection and manuscript writing.

**AU & AS:** Did review and final approval of manuscript.
